# A spectrophotometric method for determining the amount of folic acid in fortified salt

**DOI:** 10.1016/j.jafr.2020.100060

**Published:** 2020-12

**Authors:** Oluwasegun Modupe, Julie Bloquet Maurras, Levente L. Diosady

**Affiliations:** a Department of Chemical Engineering and Applied Chemistry, University of Toronto, Canada; b AgroSup Dijon, Dijon Cedex, France

## Abstract

Analytical methods for quantifying and monitoring the degradation of micronutrients added to food are crucial to food fortification programs. In the case of folic acid in fortified salts, there are difficulties in developing an effective analytical method due to interference of salt in the standard HPLC methods, as salt precipitates in the HPLC column. To circumvent the problem, a spectrophotometric method was developed to quantify folic acid and monitor its degradation in salt. A distinct absorption wavelength was selected for folic acid in sodium carbonate solution. Of the three wavelengths where maximum absorption was observed for folic acid, 285 nm was selected as being selective for folic acid in the presence of pteroic acid, glutamic acid, aminobenzoic acid, and other products of degradation of folic acid. The method was calibrated for 1-25 μg/mL folic acid (R^2^ = 1). The recovery was 100 ± 1.2% and 100 ± 1.8% for folic acid in salt and solution, respectively. The limit of detection and quantification for this method is 0.011 μg/mL and 0.033 μg/mL, respectively. The method is accurate, precise, and selective for folic acid in the presence of potential products of folic acid degradation, and is suitable for monitoring folic acid degradation in fortified salt.

## Highlights

• Folic acid in fortified salt is difficult to analyse in fortified salt matrix using the established method.
• A spectrophotometric method using 285nm that is distinct for folic acid was used to determine folic acid in fortified salt.
• 285nm was selective for folic acid in the presence of components of the fortified salt and folic acid degradation products.

## Graphical abstract

**Figure:**
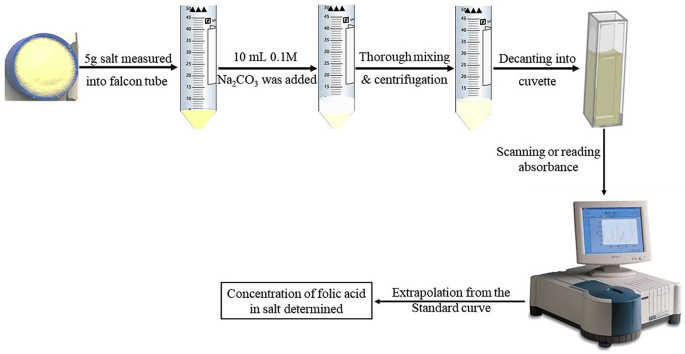

## 1 Introduction

Folates, the natural form of folic acid, is present in fruits, green vegetables, yeast, and liver. However, folates in food are quickly degraded, and there is limited access to folic acid-rich foods in some countries (Crider, Bailey and Berry, 2011). More so, it is difficult, if not impossible, to find any food that can supply the RDI (0.4 mg) of folate per serving (Exler, 2012). This explains why in most of the countries that have national data, the national prevalence is higher than 20% in low-income countries but less than 5% in high-income countries (Rogers et al., 2018). The synthetic folate, folic acid, is more stable and readily absorbed in the GIT.

The consequences of folate deficiency include neural tube defects (NTDs), megaloblastic anemia, and hyperhomocysteinemia. These consequences are worsened by the rapid depletion of folate stored in the human body [1]. Given these consequences, the USA and Canada mandated compulsory fortification of staple food with folic acid, the vitamin B_9_ fortificant [2,3]. However, mandatory fortification often comes at an increased cost, and may not be affordable for developing countries [4]. To reach vulnerable populations that may not have access or cannot afford folic acid fortified foods, Modupe et al. [5] developed a technology for the delivery of folic acid and other micronutrients through salt. The fortified salt can be made by spraying a solution of folic acid and iodine on salt and admixing an encapsulated iron premix [5]. Vitamin B_12_ was also added to the salt to complement the effect of folic acid. This salt can reduce cases of coexisting iron, iodine, folic acid, and vitamin B_12_ deficiencies that aggravates anaemia and other prenatal and postnatal complications.

**Fig. 1:**
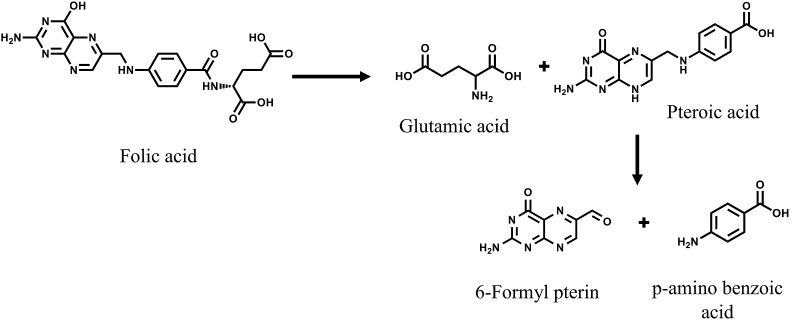
Thermal degradation of folic acid (Adapted from Ref. [5]).

Folic acid is a pteroyl monoglutamic acid. It consists of three moieties: a pteridine ring, *p*-aminobenzoate, and glutamic acid. The *p*-aminobenzoate moiety links the pteridines ring and the glutamate moiety. The stability of folic acid is affected by pH, temperature, light, oxidizing, and reducing agents. Folic acid is typically degraded by cleaving at the C9-N10 to produce 6-formylpterin and *p*-aminobenzoyl-l-glutamic acid [6]. In the case of thermal degradation, which is more likely in fortified salt [5], folic acid is degraded to glutamic acid and pteroic acid (Fig. 1). The pteroic acid is further degraded to 6-formylpterin and aminobenzoic acid [7,8].

Critical to the adoption of the folic acid fortification technology is an analytical method for quantifying folic acid and monitoring degradation of folic acid in the salt and spray solution [9]. Hence, there is a need to have a robust analytical method for quantifying folic acid added to both the spray solution and salt. The analytical method must be sensitive and selective to distinguish folic acid from its degradation products. The analytical method should enable the monitoring of how folic acid degrades in potassium iodate solution and fortified salt samples. For effective fortification programs in developing countries, the method must be inexpensive and simple.

Although there are many analytical methods for folic acid [10,11], most of the available methods are either very sophisticated or may not be applicable for the fortified salt. The extraction of folic acid from salt is challenging, given that folic acid is only very soluble in solution of sodium hydroxide and sodium carbonate [12], where salt is also soluble. Hence, there is concern that salt may precipitate in the column if an HPLC method is used. Given the amount of folic acid added to salt, the use of spectrophotometric method that does not use sodium hydroxide or sodium carbonate solution may not work for salt. The use of phosphate buffer as solvent system for extracting folic acid from pharmaceutical dilution as described by Ribeiro et al. [13] did not work for salt matrix due to the concentration of folic acid in the fortified salt. More so, there was concern that microbial analysis will be impaired by the high concentration of salt [14]. Additionally, the method for the determination of folic must be very simple and require simple and relatively cheap equipment for use in settings where salt fortification will be practiced.

Initially, the method developed by Nagaraja et al. [15] was modified for quantifying folic acid in the salt matrix. This method was based on the reductive cleavage of folic acid and the formation of a color complex of the product of folic acid degradation. However, salt or other components of the fortified salt interfered with the formation of the color complex. This study aims to develop a spectrophotometric method for quantifying folic acid (a vitamin B_9_ fortificant) in solutions and fortified salts without the interference of the products of degradation of folic acid and other constituents of these matrices.

## 2 Experimental

### 2.1 Materials

Folic acid (≥97%), vitamin B_12_, pteroic acid, glutamic acid, and *para*-aminobenzoic acid were obtained from *Sigma-Aldrich Canada (Oakville, Ontario, Canada)*. Hydrochloric acid, sodium carbonate, and zinc granules were obtained from *Caledon Laboratories Ltd, (Georgetown, Ontario, Canada).* Spray solution, used for adding folic acid and iodine to salt, was prepared by dissolving 1 g of folic acid and 3.37 g of potassium iodate in 0.1 M sodium carbonate (100 mL). Extruded and encapsulated ferrous fumarate was added to the salt already fortified with folic acid and iodine to form the Triple Fortified Salt (TFS) that was used in this study [5]. All the chemicals except those used for making the fortified salt were analytical grade.

### 2.2 Preparation of folic acid solution

A solution of sodium carbonate (0.1 M) was prepared using reverse osmosis (RO) water (pH = 11.1). This was then used to prepare 250 μg/mL folic acid (stock solution). The stock solution was diluted to 125, 100, 75, 25, and 10, 5, 2.5, and 1 μg/mL with the sodium carbonate solution. Sodium carbonate was used because it is part of the iodine and folic acid solution that is sprayed on salt when TFS is formulated.

### 2.3 Scanning the absorption wavelength for folic acid

The absorbance of the 25 μg/mL folic acid solution was scanned between 240 and 800 nm, using a Cary-50 UV⁄Vis spectrophotometer(Varian Inc. CA, USA). The sodium carbonate solution was used as the baseline solution. The scanning speed was 240 nm/min and the Hellma® absorption cuvettes with 1 cm pathlength were used. For accuracy and precision of the scanning protocol, two separate 25 μg/mL folic acid solutions were scanned twice each.

### 2.4 Evaluation of the specificity of the method

The method described by Nagaraja et al. [15] was used to reductively cleave folic acid to pterine-6-carboxylic acid (PCA) and *p*-aminobenzoyl-l-glutamic acid (pABGA). The reaction was designed such that the starting concentration of folic acid was 25 μg/L. Three identical reaction setups were made - the first was scanned (240 -500 nm) after 30 min, the second after 60 min, and the third after 120 min of degradation reaction. Also, 10 μg/mL pteroic acid, 10 μg/mL *para*-aminobenzoic acid, 10 μg/mL glutamic acid, 10 μg/mL cyanocobalamin and 10 μg/mL folic acid solutions were scanned (230-700 nm). The distinct wavelength for folic acid was identified using the data obtained from all these scans.

Also, the selectivity of the analytical method for folic acid spray solution in the presence of other possible components of the spray solution and salt was evaluated. The absorbance of solutions containing 10 ​μg/mL folic, 10 ​μg/mL folic acid +20 ​μg/mL iodine, and 10 ​μg/mL folic acid +20 ​μg/mL iodine +10 ​μg/mL citrate was measured. The concentrations were based on the proportions of the constituents that would be extracted from salt.

The selectivity of the analytical method for folic acid in the presence of the product of reductively cleaved folic acid was probed. Folic acid solution (2.5 μg/mL and 25 μg/mL) were subjected to reductive cleavage, using 5 N hydrochloric acid and zinc [15]. The absorbance of the solutions before and after the reaction was measured at the chosen wavelength of 285 nm.

### 2.5 Determination of the linear range

The stock solution was diluted to produce 125, 100, 75, 25, 10, 5, 2.5, and 1 μg/mL standards. The linearity of the absorbance between 2.5 and 250 μg/mL was first evaluated. This was repeated between 1 and 50 μg/mL. The effect of the use of sodium carbonate solution compared to the use of water to dilute the stock solution was evaluated.

### 2.6 Extraction of folic acid from spray solution and salt

For salt, 5g of salt was weighed into a 50 mL falcon tube, and 10 mL of 0.1 M sodium carbonate solution was added. The mixture was thoroughly mixed for 1 min. The supernatant was filtered with a 0.45 μm syringe filter., and the absorbance of the filtrate was read. For spray solutions, 100 μL of the solution was measured with a 10-100 μL micropipette and diluted to 100 mL with 0.1 M sodium carbonate in a volumetric flask. The absorbance of the solution was then read. In order to determine the impact of light on the method, extracted folic acid was stored in a transparent scintillation vial exposed to the natural laboratory light for 5 h before its absorbance measured.

### 2.7 Determination of limit of detection and quantification

The limits of detection and quantification of folic acid in salt were calculated using the expressions as described by ICH [16] Guideline, as expressed in Equation (1)&2.

(1) $$LOD=\frac{3.3\;\sigma }{S}$$

(2) $$LOQ=\frac{10\sigma }{S}$$

Where σ = standard deviation of the response of blank at the chosen wavelength, and
S = slope of the calibration curve

### 2.8 Percentage recovery

The percentage recovery from spray solution and fortified salt was evaluated as expressed in Equation (3). Based on the linear regression equation of the calibration curve, the absorbance of folic acid extracted from salt and spray solution was measured on different days and in two different laboratories using different spectrophotometers of the same make and model

(3) $$\%\;Recovery=\frac{Amount\;of\;folic\;acid\;analyzed\;in\;salt\;or\;solution}{Amount\;of\;folic\;acid\;added\;to\;salt\;or\;solution\;\;}\times 100$$

The absorbance of folic acid extracted from salt was repeatedly measured at 285 nm over 5 h.

### 2.9 Statistical analysis

The results were expressed as the mean ± Standard error of mean (SEM) of at least six determinations. The data were analyzed using Duncan Multiple Range Test. The differences were considered statistically significant at p < 0.05. All the analyses were done using SPSS version 16.0 software (SPSS Inc., Chicago, IL, USA).

## 3 Results and discussion

An analytical method that is accurate and selective for folic acid in the presence of other component of salt matrix (especially products of degradation of folic acid and other components of salt) is crucial for rapid prototyping and technology development focusing on adding folic acid to salt and other food matrices. A modification of the method developed by Nagaraja et al. [15] was initially used for the quantification of folic acid. This method is based on the catalytic and reductive cleavage of folic acid with zinc granules and hydrochloric acid, diazotization of the cleaved product with nitrite, and coupling with 3-amino phenol. However, the method was not reproducible in salt. As the method involves many reaction steps, there was room for multiple errors. Hence, we developed a method, which is relatively simple and fast, based on the distinct absorption pattern of folic acid.

**Fig. 2:**
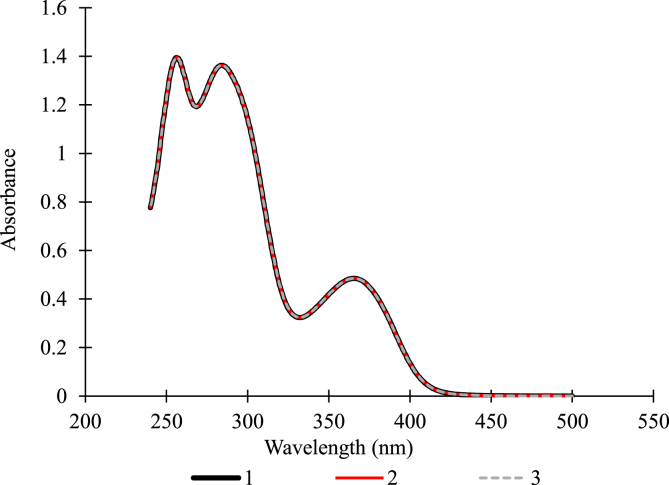
The absorption spectra of 25 μg/mL folic acid (3 independent replicates).

The first step in determining the absorption wavelength that is distinct for folic acid was the scanning of the absorbance of 25 μg/mL folic acid over a range of wavelength (230-600 nm). Maximum absorption were observed at **256 nm, 283 nm, and 366 nm** (Fig. 2). Although the wavelengths observed were slightly different from those observed by Matias et al. [17], they also reported three wavelenghts for folic acid in a phosphate buffer. The scanning procedure was precise and accurate. The same spectra were obtained when two solutions of 25 μg/mL folic acid were scanned, and when a solution of 25 μg/mL folic acid was scanned twice (Fig. 2).

**Fig. 3:**
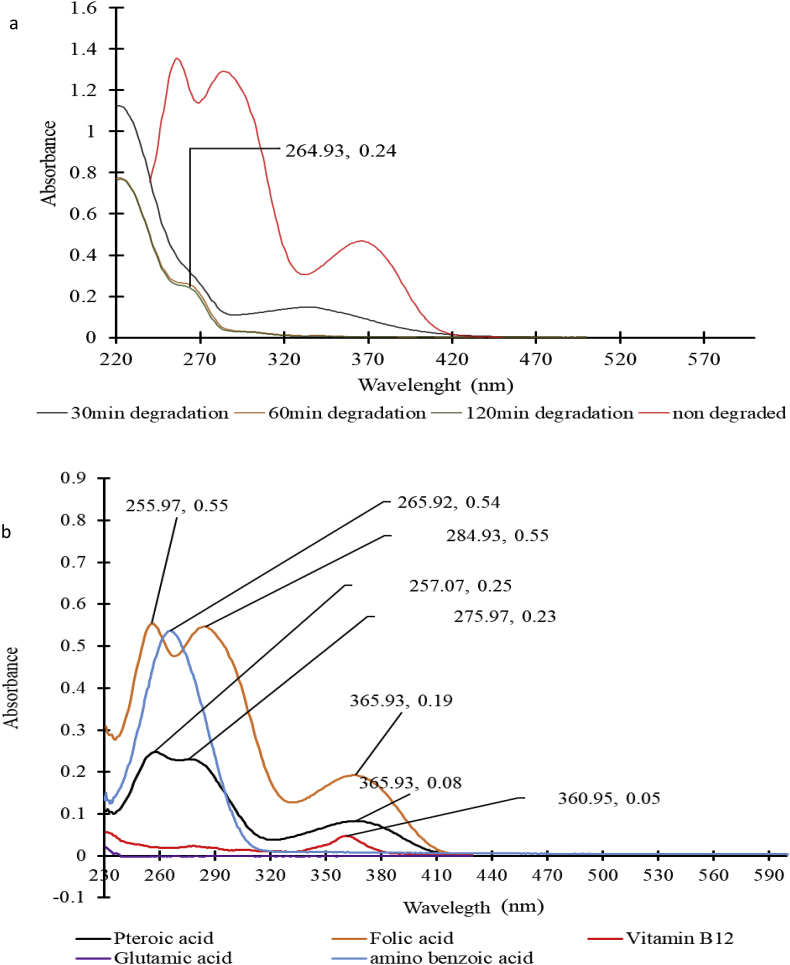
Comparative absorption spectra of folic acid and its potential products of degradation. (a) Folic acid degraded for 30, 60 & 120 min by the method described by Nagaraja et al. (2002) (b) solution of the pure potential product of degradation of folic acid.

The choice of one of the three absorption wavelengths was based on selectivity for folic acid in the presence of its degradation products and other components of salt. To make this choice, folic acid degraded to p-amino benzoyl glutamic acid, and pterine-6-carboxylic acid by the method described by Nagaraja et al. [15] was scanned. Also, the solutions (10 μg/mL) of the potential products of degradation of folic acid (pteroic acid, *para*-aminobenzoic acid, and glutamic acid) and vitamin B_12_ were scanned. At 285 nm, the maximum absorption was significantly reduced with the time of degradation of folic acid (Fig. 3a). This result is consistent with the study reported by Gazzali et al. [6]. Folic acid shared similar maximum absorption with pteroic acid at 256 and 365 nm and with vitamin B_12_ at 365 nm. Aminobenzoic acid had a distinct maximum absorption at 266 nm, which was similar to the maximum absorption observed with the degraded folic acid (Fig. 3b). These observations led to the choice of 285 nm as the distinct wavelength for folic acid.

The selectivity of the chosen wavelength (285 nm) to distinguish the degradative product of folic acid was evaluated (Table 1). The absorbance of 2.5 and 25 μg/mL of folic acid degraded by the method described by Nagaraja et al. [15] was measured. 2.5 and 25 μg/mL were selected because of the concentration of folic acid in fortified salt (25 ppm) and the possibility of losing 90% of the folic acid added to salt. No absorbance was observed when 2.5 μg/mL folic acid was degraded. Even with a higher concentration (25 μg/mL), only negligible absorbance was observed (0.25 ± 0.00) compared with an absorbance of 1.32 ± 0.00 observed for the absorbance of non-degraded folic acid (25 μg/mL). This absorbance may be attributed to remaining folic acid, not cleaved under the reaction conditions. Still, this showed that the method could distinguish between folic acid and the product of the reductive cleavage of folic acid.

**Table 1:** Selectivity of the analytical method to the product of degradation of folic acid.

Concentrations of Folic Acid (μg/mL)	Absorbance at 285 nm	
	Folic Acid	Reductively Cleaved Folic Acid
2.5	0.13 ± 0.00	−0.03
25	1.32 ± 0.00	0.25 ± 0.00

Data were expresed as mean ± standard deviation of 6 sample size.

The selectivity of the wavelength for folic acid in the presence of potential constituents of salt was evaluated. The levels of the other components in the solution represented their ratio to folic acid in the spray solution or salt. There was no significant change in the absorbance of folic acid in the presence of citrate, potassium iodate, and or vitamin B_12_ (Table 2). This showed that the other constituents would not interfere with the absorbance of folic acid at 285 nm.

**Table 2:** Selectivity of the 285 nm to folic acid in the presence of other components of salt.

Folic acid (μg/mL)	Absorbance at 285 nm			
	Folic Acid	Folic Acid ​+ ​Iodine	Folic Acid ​+ ​Iodine ​+ ​B_12_	Folic Acid ​+ ​Iodine ​+ ​B_12_ ​+ ​Citrate
10	0.5494 ± 0.0081	0.5635 ± 0.0154	0.5581 ± 0.0017	0.5544 ± 0.0026

Data were expressed as mean ± standard deviation of 6 sample size.

**Fig. 4:**
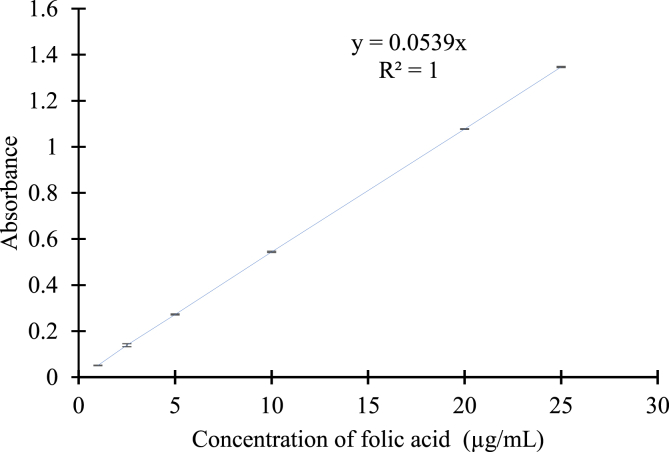
Absorbance - concentration relationship of folic acid solution in 0.1 M sodium carbonate. Data were expresed as mean ± standard deviation of 6 sample size.

A scatter plot of the concentrations and absorbance confirmed a linear relationship between absorbance and folic acid concentration in the range of 1-50 μg/mL. For increased reliability, the calibration range was narrowed to 1-25 μg/mL (Fig. 4), which reduced the maximum absorbance to less than 1.5 [18]. The linear regression equation for calculating the amount of folic acid in salt and spray solution based on the absorbance at 285 nm was obtained thus;

(15) $${\text{A}}_{285\text{nm}}=0.0539\times \left[\text{folic ​acid}\right]$$

with a correlation coefficient (R^2^) = 1.00.

The concentration of folic acid in fortified salt (12.5 or 25 μg/g) fell within the linear range when dissolved 1g of salt was dissolved in 1 mL 0.1 M sodium carbonate. This concentration is equivalent to 50-100% RDI (dietary folate equivalents). Although the concentration of folic acid in the spray solution used was higher than this range, it could be easily diluted to fall within this range. The limit of detection (LOD) and limit of quantification (LOQ) were determined based on the ICH [16] Guideline as 0.011 and 0.033 μg/mL, respectively (Table 3). The limit of quantification showed that the method could be used to quantify the amount of folic acid in fortified food, especially in salt, that is significantly lower than the RDI. The LOD and LOQ observed in this study were close to that reported by Matias et al. [17] despite the use of different solvent. The ranges of the recoveries of folic acid from salt and spray solutions were 99-101.7% and 98.2-104.1%, respectively. Hence, the analytical method has acceptable accuracy. Since the data were obtained from experiments carried out on different days and at different laboratories, the analytical method may be said to be precise. The analytical parameters are summarized in Table 3.

**Table 3:** Analytical parameters.

Parameters/Characteristics	Values
Colour of folic acid solution	Slightly yellow to colorless depending on the folic acid concentration
*λ*_max_ (nm)	285
Stability (h)	20hr
Beer's law range (μg/ml)	1–25
Limit of detection (μg/ml)	0.011
Limit of quantification (μg/ml)	0.033
Percentage recovery (salt) (%)	100 ± 1.2
Percentage recovery (spray solution) (%)	100 ± 1.9
**Regression Equation**	
Slope	0.0539
Intercept	0.00
Correlation coefficient (*R*)	1.00

The stability of the analytical method for quantifying folic acid in salt was evaluated. The stability was observed for 20 h (Table 4). After 20hr, some white precipitates were observed in the solution of folic acid extracted from salt, which interfered with the absorbance of folic acid at 285 nm. The folic solution extracted from salt was exposed to light for 5hr. The absorbance at 285 nm was the same throughout this period. Hence, a short time exposure of the light as it may occur during sample preparation should not impact the measurement.

**Table 4:** Stability of the analytical method for quantifying folic acid extracted from fortified salt.

Times (hr)	Absorbance
0	1.3467 ± 0.0044
0.5	1.3415 ± 0.0142
1	1.3435 ± 0.0132
2	1.3412 ± 0.0223
20	1.3480 ± 0.0148

Data were expressed as mean ± standard deviation of 6 sample size.

With the analytical method, the impact of the process developed for the fortification of salt on folic acid stability can be monitored, field tests of the fortified salt would be possible to monitor the impact of environmental conditions of the distribution channels on the stability of folic acid in the salt and to identify counterfeit products with fake folic acid labels. Although the analytical method is developed for quantifying folic acid in fortified salt, the method may be adapted for quantifying folic acid in other fortified foods.

## 4 Conclusion

A low-cost and simple spectrophotometric method was developed for determining the concentration of folic acid in spray solutions and triple and quadruple fortified salts containing potassium iodate, folic acid, vitamin B_12,_ and iron as encapsulated ferrous fumarate. The method is based on absorption at 285 nm, which is selective for folic acid in the presence of products of degradation of folic acid and vitamin B_12_. High recoveries of folic acid were obtained from spray solutions and salt (100 ± 1.8 and 100 ± 1.2), confirming that the method is accurate and precise. The analytical method can be used to monitor the progression of folic acid degradation in the spray solution and salt fortified by a spray solution of potassium iodate, sodium carbonate, and folic acid. The analytical method is stable and can withstand exposure to light for at least 5 h needed to complete the analysis.

## Declaration of competing interest

There are no conflicts to declare.
